# Sex Differences in the Impact of Cardiometabolic Burden on Cognition in Persons 55+

**DOI:** 10.1002/alz70857_101005

**Published:** 2025-12-24

**Authors:** Stella Garriga, Lauren G Santos, Jared Block, Brittany Wolff, Lucia Cavanagh, Robert Bilder, Laura Glass Umfleet, Adriana Strutt, Paola A Suarez, Franchesca Arias

**Affiliations:** ^1^ University of Florida, Gainesville, FL, USA; ^2^ University of California Los Angeles, Los Angeles, CA, USA; ^3^ Medical College of Wisconsin, Wauwatosa, WI, USA; ^4^ Baylor University, Waco, TX, USA; ^5^ University of California, Los Angeles, Los Angeles, CA, USA

## Abstract

**Background:**

Females with greater cardiometabolic burden (CMB) exhibit faster rates of cognitive decline compared to their male counterparts. Critical knowledge gaps exist, however, in understanding whether biological sex, a fundamental determinant of health outcomes, moderates the association between CMB and cognition differentially across cognitive domains. This is concerning given the well‐documented sex disparities in both cardiometabolic and cognitive disorders, suggesting distinct pathophysiological mechanisms. This study leverages the resources available through the National Neuropsychology Network (NNN), a multi‐site observational study, to delineate how sex and CMB interact to shape cognitive outcomes.

**Method:**

This cross‐sectional analysis included 4,258 participants enrolled in the NNN study (50.3% Female, 49.7% Male; *M_education_* = 15.2 ±  2.8; 80.0% Non‐Hispanic White; *M_age_* = 69.3 ±  8.35). We identified the most prevalent cardiometabolic conditions in the United States and used pre‐visit ICD‐10 diagnostic codes to determine the prevalence of these conditions in our sample. CMB was estimated based on the aggregated number of conditions present. Neuropsychological measures were grouped by domain and statistically confirmed via confirmatory factor analysis (CFA): working memory/attention, language, executive function, memory, visuoperceptual abilities, and processing speed. MANCOVAs were used to compare domain‐level performance across CMB groups, accounting for sex, education, race, and age.

**Result:**

The CFA supported the proposed model (*Χ* ^2^(856)=2524.135, *p* < .001; *RMSEA* =  0.046; *CFI* = 0.916; *TLI* = 0.909). There was a main effect of CMB on reduced performance in executive function (*F*(2,1569)=6.14, *p* < .01), processing speed (*F*(2, 726)=12.98, *p* < .001), and language (*F*(3,952)=3.96, *p* < .01). Female sex was associated with reduced performance in working memory/attention (*F*(2,733)=25.61, *p* < .001), executive function (*F*(2,1569)=5.72, *p* < .01), processing speed (*F*(2,726)=5.81, *p* < .01), and visuoperceptual abilities (*F*(2,464)=26.15, *p* < .001). The interaction effect between sex and CMB was significant for visuoperceptual abilities (*F*(2,464)=5.66, *p* < .01).

**Conclusion:**

Sex and increasing CMB differentially predicted domain‐level cognitive deficits, with female sex conferring increased vulnerability in visuoperceptual abilities. While cardiometabolic conditions were similarly prevalent in both sexes, their impact on cognition varied. These results underscore the importance of reducing CMB to promote brain health and the need for tailored cognitive assessment and intervention. Future research should investigate underlying mechanisms of noted sex differences, including potential hormonal influences, vascular factors, and sex‐specific inflammatory responses.

